# *Plasmodium falciparum* infection prevalence among children aged 6–59 months from independent DHS and HIV surveys: Nigeria, 2018

**DOI:** 10.1038/s41598-023-28257-0

**Published:** 2023-02-03

**Authors:** Adan Oviedo, Ado Abubakar, Perpetua Uhomoibhi, Mark Maire, Uwem Inyang, Bala Audu, Nnaemeka C. Iriemenam, Abiodun Ogunniyi, James Ssekitooleko, Jo-Angeline Kalambo, Stacie M. Greby, Nwando Mba, Mahesh Swaminathan, Chikwe Ihekweazu, McPaul I. Okoye, Eric Rogier, Laura C. Steinhardt

**Affiliations:** 1 Malaria Branch, Division of Parasitic Diseases and Malaria, United States Centers for Disease Control and Prevention, Atlanta, GA 30029 USA; 2 Institute of Human Virology Nigeria, Abuja, Nigeria; 3 National Malaria Elimination Programme, Federal Ministry of Health, Abuja, Nigeria; 4 US President’s Malaria Initiative, Abuja, Nigeria; 5 United States Agency for International Development, Abuja, Nigeria; 6 Division of Global HIV and TB, US Centers for Disease Control and Prevention, Abuja, FCT Nigeria; 7 Nigeria Centre for Disease Control, Abuja, Nigeria; 8 The Global Fund to Fight AIDS, Tuberculosis, and Malaria, Geneva, Switzerland

**Keywords:** Policy and public health in microbiology, Diagnostic markers, Infectious-disease diagnostics

## Abstract

Prevalence estimates are critical for malaria programming efforts but generating these from non-malaria surveys is not standard practice. Malaria prevalence estimates for 6–59-month-old Nigerian children were compared between two national household surveys performed simultaneously in 2018: a Demographic and Health Survey (DHS) and the Nigeria HIV/AIDS Indicator and Impact Survey (NAIIS). DHS tested via microscopy (n = 8298) and HRP2-based rapid diagnostic test (RDT, n = 11,351), and NAIIS collected dried blood spots (DBS) which were later tested for histidine-rich protein 2 (HRP2) antigen (n = 8029). National *Plasmodium falciparum* prevalence was 22.6% (95% CI 21.2– 24.1%) via microscopy and 36.2% (34.6– 37.8%) via RDT according to DHS, and HRP2 antigenemia was 38.3% (36.7–39.9%) by NAIIS DBS. Between the two surveys, significant rank-order correlation occurred for state-level malaria prevalence for RDT (Rho = 0.80, p < 0.001) and microscopy (Rho = 0.75, p < 0.001) versus HRP2. RDT versus HRP2 positivity showed 24 states (64.9%) with overlapping 95% confidence intervals from the two independent surveys. *P. falciparum* prevalence estimates among 6–59-month-olds in Nigeria were highly concordant from two simultaneous, independently conducted household surveys, regardless of malaria test utilized. This provides evidence for the value of post-hoc laboratory HRP2 detection to leverage non-malaria surveys with similar sampling designs to obtain accurate *P. falciparum* estimates.

## Introduction

Prevalence estimates from cross-sectional surveys and surveillance programs have a foundational role in guiding management of infectious disease control programs. Employing accurate, efficient, and robust tools to estimate prevalence on a large geographic scale becomes especially crucial in heterogeneous settings, as is the case in many countries endemic for *Plasmodium *sp., the parasites responsible for malaria^1–4^. Various malaria transmission metrics are currently used, including vector-based measures such as the entomological inoculation rate (EIR) and vectorial capacity, and parasite-based measures including parasite prevalence, incidence of clinical malaria, force of infection, seroprevalence, and seroconversion rate^5^. A common measure of malaria burden in the populace is parasite prevalence, obtained through national household surveys such as the Demographic and Health Surveys^6^, and other specialized surveys designed to measure the effects of malaria interventions or similar metrics^4^. Survey-based measures of parasite prevalence in a target population should yield similar estimates if done during the same transmission season and using similar methods, although estimated prevalence can vary widely depending on the diagnostic test used, e.g., rapid diagnostic test (RDT), light microscopy (LM), or molecular-based methods (PCR)^7,8^. However, limited evidence exists of this concordance in estimates from different independent surveys conducted during the same time period in the same area but using different methods.

While LM is a method of visually detecting blood-stage parasites and is considered the gold standard for parasite identification, it requires well-trained technicians, routine maintenance, and infrastructure which are not always available^7,9–11^. RDTs detect *Plasmodium *sp. antigens in whole blood and are less challenging to perform and interpret in low-resource settings^7,9,10,12,13^. The *P. falciparum* specific histidine-rich protein 2 (HRP2) may be present in the host weeks after parasite clearance, meaning that a positive RDT may be measuring HRP2 antigen from either a current or recent infection^14,15^. PCR provides increased sensitivity but is not easily accessible in low-resource settings due to the high cost and more complex instrumentation needed^16^. More recently, advancements in antigen detection in the laboratory have produced multiplex assays which can detect picogram levels of *Plasmodium* antigens^17,18^. For the bead-based multiplex assay, multiple sample types have been validated, including dried blood spots (DBS)^19^.

The 2018 Nigeria Demographic and Health Survey (DHS)^20^ and the Nigeria HIV/AIDS Indicator and Impact Survey (NAIIS)^21^ were large cross-sectional household surveys both occurring in the latter half of 2018 across Nigeria. The DHS primary objective was to provide information on demographic and health indicators throughout Nigeria, which included malaria prevalence. The NAIIS primary objectives were to estimate incidence and prevalence of HIV and viral load suppression on a population level, and DBS samples were prepared from blood collected from consenting individuals for later laboratory analyses as part of the Nigeria Multi-disease Serologic Surveillance using Stored Specimens (NMS4) project.

This report compares malaria prevalence estimates generated by the DHS and NMS4 among 6–59-month-old children in Nigeria. Although conducted separately and independent of each other, both cross-sectional surveys occurred during the same six-month period of time, and both utilized survey designs representing the same population. This unique opportunity allows for comparing national and state-level malaria prevalence estimates from two independent surveys to investigate concordance in quantitative estimates by geographic locality, time period, and other variables. The findings presented here serve as an example of utilizing samples collected for another disease program to obtain accurate malaria estimates.

## Results

### Survey populations

Of the 41,688 households selected for inclusion from all 36 states and the FCT by the DHS, 40,666 were occupied and 40,427 provided responses, yielding a household response rate of 99.4%. Of the total eligible children enrolled in the DHS, 96.9% were successfully tested by HRP2-based RDT and 96.3% via microscopy, yielding a total of 11,173 and 8127 tested using RDT and microscopy, respectively (Supplementary Table 1). During the same period in 2018, 101,267 households were selected for inclusion into NAIIS. Of 89,345 occupied homes, 83,909 provided responses yielding a household response rate of 93.9%. A total of 205,903 individuals of all ages and from all 36 states and FCT of Nigeria consented to the storage of specimens with future testing. Among the households selected, the blood draw response rate among children 0–9 years was 68.5%, and bead antigen assay data were obtained from 8029 children between the ages of 6–59-months.

**Figure 1:**
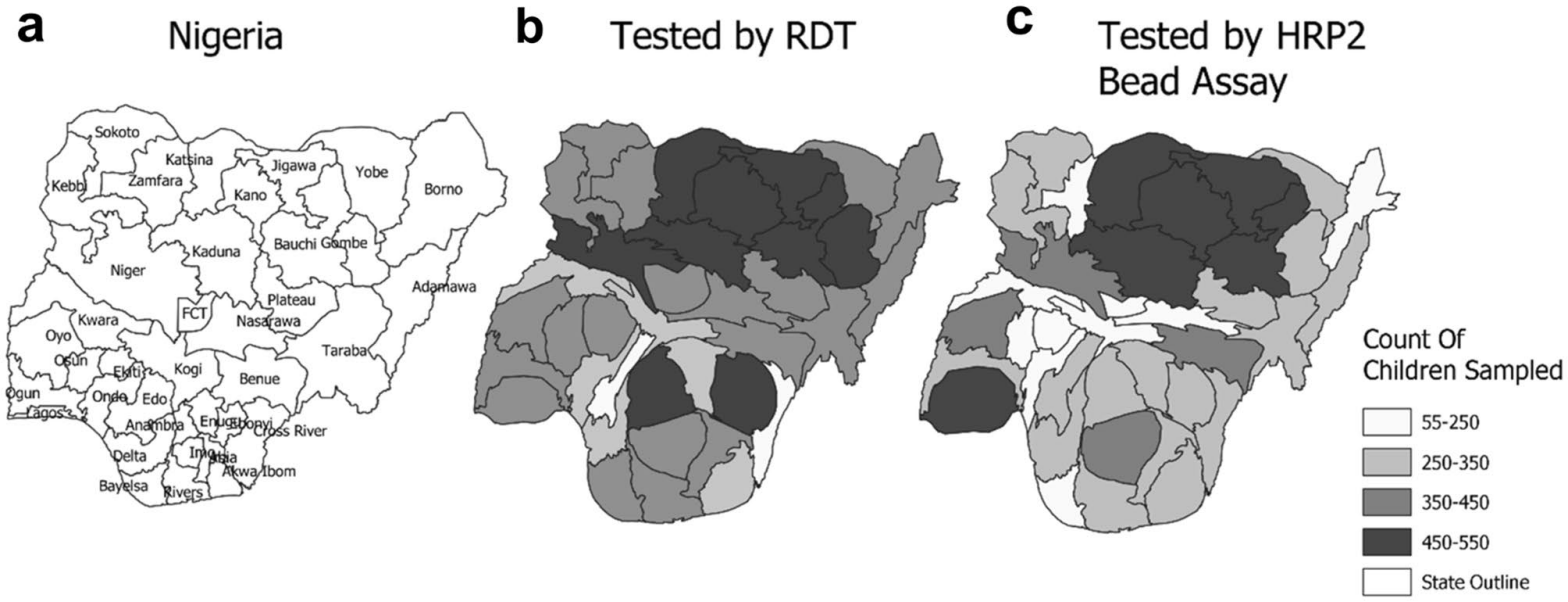
Test results as available for Nigerian states and FCT. Geopolitical state boundaries of Nigeria (**a**) and cartograms illustrating unweighted counts of children with test results for RDT from the DHS (**b**) and HRP2 bead assay from NAIIS samples (**c**). Maps were generated using QGIS version 3.12.2 (www.qgis.org).

Roughly half the population surveyed were girls for both the DHS (49.5%) and NMS4 (48.7%) (Supplementary Table 1). Children in the 6–23 months age group represented 33.6% of children surveyed in the DHS (mean age 32.2 months) compared to 15.6% in the NMS4 (mean age 32.2 months). While the two most sampled states in DHS by total children via RDT were Kano (4.6%) and Ebonyi (4.0%), the top two most sampled states in the NMS4 were Kaduna (6.0%) and Katsina (5.1%) (Fig. 1b,c). By month, the largest percentage of children sampled in the DHS via microscopy (28.6%) and RDT (28.4%) was in September, while the largest percentage in the NMS4 was in October (21.1%). However, NAIIS data were collected during a period of less than one month in each state, while the DHS spread out data collection in most states throughout the five-month survey period.

### Prevalence estimates

According to the DHS, nationwide *P. falciparum* prevalence among 6–59-month-old children was 22.6% (95% CI 21.2–24.1%) by microscopy and 36.2% (95% CI 34.6–37.8%) by RDT. NMS4 *P. falciparum* prevalence among the same population was 38.3% (95% CI 36.7–39.9%) via bead-based HRP2 assay (Table 1). Prevalence estimates increased with age by all testing methods. The largest difference in prevalence by age between the DHS and NMS4 survey estimates was noted among the 6–23-month-old children, with a prevalence of 28.9% (95% CI 27.0–30.8) for RDT compared to a prevalence of 23.2% (95% CI 20.5–25.9) for HRP2 bead assay (Table 1). Prevalence differences were within 3% for all other age groups.

**Table 1:** Weighted malaria prevalence estimates and 95% confidence intervals between DHS and NMS4 by demographic and geographic variables among 6–59-month-old children in Nigeria, 2018.

	DHS		NMS4
	Microscopy	RDT	HRP2 bead assay
	Prevalence (95% CI)	Prevalence (95% CI)	Prevalence (95% CI)
Total	22.6 (21.2–24.1)	36.2 (34.6–37.8)	38.3 (36.7–39.9)
Sex			
Female	21.8 (20.0–23.6)	35.7 (33.9–37.6)	37.7 (35.7–39.7)
Male	23.4 (21.7–25.2)	36.6 (34.7–38.6)	39.0 (37.0–41.0)
Age in months			
6–23	17.9 (16.1–19.7)	28.9 (27.0–30.8)	23.2 (20.5–25.9)
24–35	20.1 (17.8–22.5)	36.6 (34.1–39.1)	35.3 (32.8–37.9)
36–47	24.3 (21.9–26.7)	40.5 (37.9–43.2)	41.2 (38.7–43.6)
48–59	30.8 (28.2–33.5)	42.7 (40.2–45.3)	45.2 (42.6–47.7)
Month of collection			
July	–	–	29.3 (23.3–35.4)
August	14.5 (9.2–19.8)	26.2 (19.5–32.9)	31.5 (28.1–34.9)
September	25.1 (22.1–28.1)	38.3 (34.9–41.6)	39.4 (35.5–43.2)
October	23.3 (20.2–26.3)	37.6 (34.0–41.3)	48.6 (45.4–51.8)
November	22.0 (18.9–25.2)	36.8 (33.2–40.4)	42.2 (39.0–45.3)
December	22.2 (17.9–26.5)	35.0 (30.6–39.5)	35.1 (31.0–39.2)
State			
North Central	21.2 (18.0–24.5)	37.0 (33.1–40.9)	41.0 (37.1–44.9)
Benue	12.7 (8.5–17.0)	26.0 (19.2–32.8)	31.0 (22.4–39.7)
FCT	20.1 (13.2–26.9)	31.3 (22.1–40.5)	22.1 (10.7–33.5)
Kogi	25.4 (16.2–34.6)	46.0 (35.0–56.9)	46.0 (35.4–56.7)
Kwara	20.2 (12.3–28.2)	43.7 (30.0–57.4)	44.7 (28.9–60.6)
Nasarawa	13.6 (7.2–20.1)	32.1 (22.9–41.4)	46.5 (34.1–58.8)
Niger	31.6 (22.5–40.7)	43.8 (35.9–51.7)	51.3 (44.0–58.6)
Plateau	21.4 (12.7–30.2)	37.2 (25.5–48.9)	31.9 (23.3–40.5)
North East	19.9 (16.8–22.9)	35.6 (32.3–38.9)	41.8 (37.6–46.0)
Adamawa	21.1 (12.8–29.5)	38.9 (28.7–49.0)	52.8 (44.4–61.1)
Bauchi	30.6 (21.9–39.2)	48.6 (40.8–56.3)	46.1 (38.4–53.7)
Borno	10.0 (5.5–14.5)	16.2 (10.5–21.8)	23.5 (11.9–35.2)
Gombe	30.3 (21.7–39.0)	52.0 (43.1–60.8)	51.3 (41.4–61.1)
Taraba	20.8 (15.0–26.7)	35.2 (28.2–42.1)	49.4 (40.5–58.2)
Yobe	13.3 (7.5–19.1)	30.3 (23.5–37.2)	26.7 (18.5–34.9)
North West	33.8 (30.4–37.1)	49.5 (46.1–52.9)	49.6 (46.2–52.9)
Jigawa	35.7 (29.0–42.4)	49.4 (43.0–55.8)	51.6 (44.4–58.8)
Kaduna	33.0 (24.6–41.4)	34.3 (26.5–42.1)	58.2 (49.9–66.4)
Kano	32.4 (25.0–39.8)	43.0 (35.3–50.7)	33.9 (26.2–41.5)
Katsina	25.5 (18.4–32.5)	55.4 (47.5–63.3)	52.2 (45.3–59.2)
Kebbi	52.2 (43.1–61.3)	76.8 (70.1–83.5)	71.8 (64.2–79.4)
Sokoto	36.4 (26.8–45.9)	54.7 (45.5–63.8)	63.8 (56.4–71.3)
Zamfara	35.7 (23.7–47.8)	51.8 (40.6–62.9)	43.9 (34.4–53.4)
South East	15.7 (12.3–19.0)	26.1 (22.4–29.8)	33.3 (29.9–36.7)
Abia	13.5 (6.4–20.5)	20.7 (13.0–28.4)	24.3 (16.3–32.4)
Anambra	8.8 (4.2–13.5)	15.2 (10.0–20.3)	14.9 (9.0–20.7)
Ebonyi	30.5 (22.6–38.5)	49.3 (41.6–57.1)	54.1 (47.0–61.3)
Enugu	17.4 (8.7–26.2)	30.2 (20.3–40.0)	35.8 (27.3–44.2)
Imo	7.8 (2.5–13.1)	15.6 (9.5–21.7)	22.1 (16.1–28.0)
South South	15.6 (12.0–19.3)	25.4 (21.7–29.2)	27.5 (24.2–30.8)
Akwa Ibom	23.2 (14.5–31.9)	33.2 (26.1–40.3)	44.3 (36.2–52.3)
Bayelsa	12.5 (8.1–17.0)	30.1 (22.8–37.4)	26.6 (16.8–36.4)
Cross River	19.5 (6.9–32.0)	26.4 (15.0–37.7)	48.0 (38.2–57.9)
Delta	17.0 (6.6–27.5)	24.9 (14.7–35.2)	26.5 (19.1–33.9)
Edo	14.7 (7.0–22.5)	19.1 (10.5–27.6)	29.2 (20.7–37.6)
Rivers	11.2 (4.8–17.5)	22.3 (14.9–29.8)	25.6 (18.3–32.9)
South West	18.4 (15.2–21.6)	28.9 (25.3–32.5)	22.4 (19.4–25.3)
Ekiti	32.3 (23.0–41.6)	46.3 (36.5–56.2)	50.3 (38.7–61.9)
Lagos	1.8 (0.1–3.4)	3.4 (1.3–5.5)	3.5 (1.5–5.4)
Ogun	21.6 (14.2–29.0)	32.2 (22.7–41.7)	22.7 (13.6–31.8)
Ondo	33.5 (24.1–42.9)	41.6 (29.2–54.0)	38.6 (27.2–50.0)
Osun	27.7 (20.7–34.8)	54.9 (46.4–63.5)	47.4 (35.8–59.0)
Oyo	23.8 (14.8–32.7)	33.9 (26.4–41.5)	42.5 (34.0–51.0)

*DHS* Demographic Health Survey, *FCT* Federal Capital Territory, *HRP2* Histidine-rich protein 2, *NMS4* Nigeria Multi-disease Serologic Surveillance using Stored Specimens, *RDT* rapid diagnostic test.

**Figure 2:**
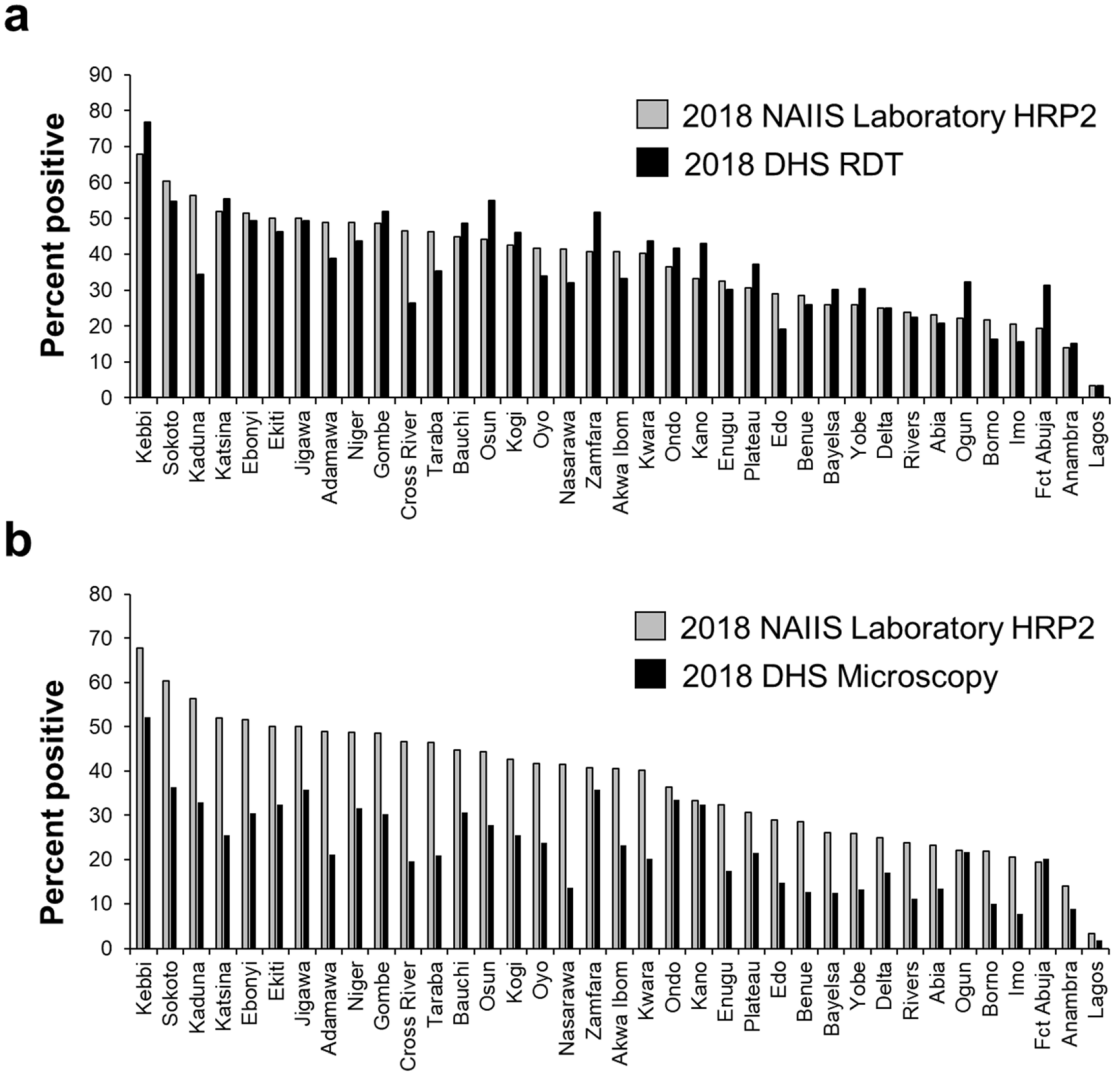
Malaria antigen prevalence by Nigerian state among 0–59-month-olds from 2018 NAIIS compared to the 2018 DHS. (**a**) RDT prevalence by DHS compared with HRP2 antigen prevalence from NAIIS samples. (**b**) Microscopy prevalence by DHS compared with HRP2 antigen prevalence from NAIIS samples.

**Figure 3:**
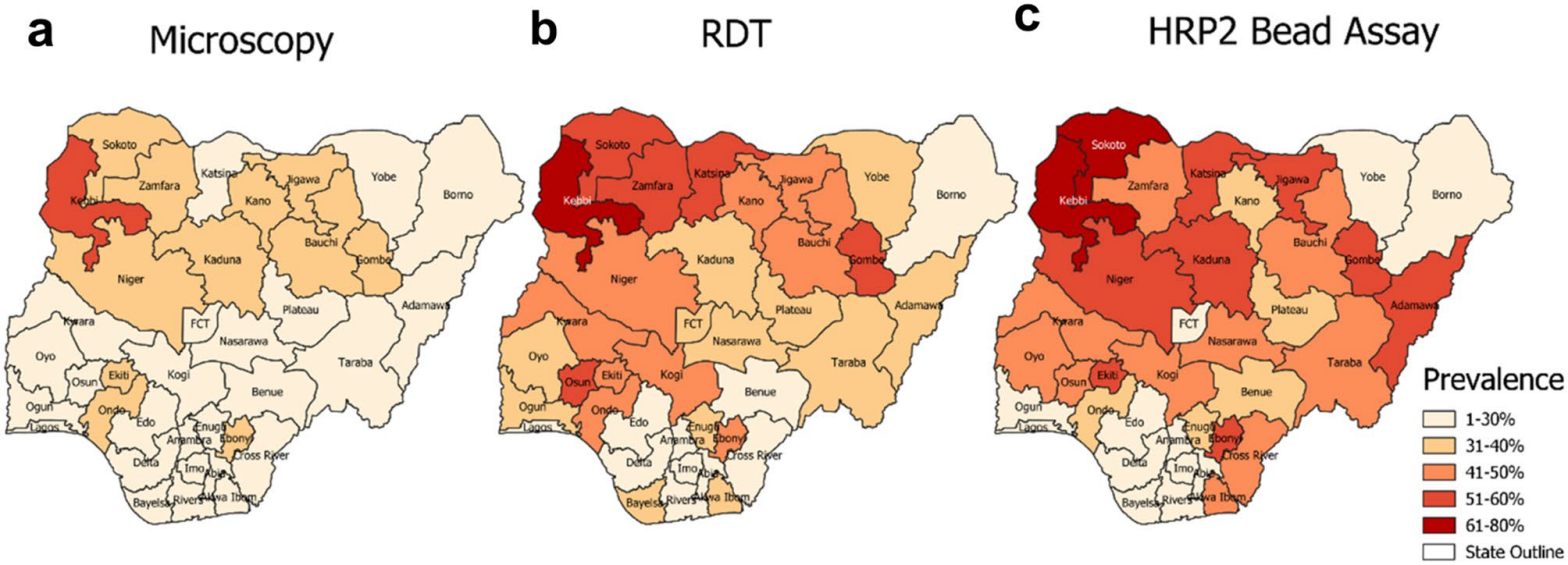
*P. falciparum* infection prevalence estimates by Nigerian state among children 6–59 months old. Results by microscopy (**a**) and RDT (**b**) from the 2018 DHS and HRP2 bead assay (**c**) from the 2018 NAIIS. Maps were generated using QGIS version 3.12.2 (www.qgis.org).

**Figure 4:**
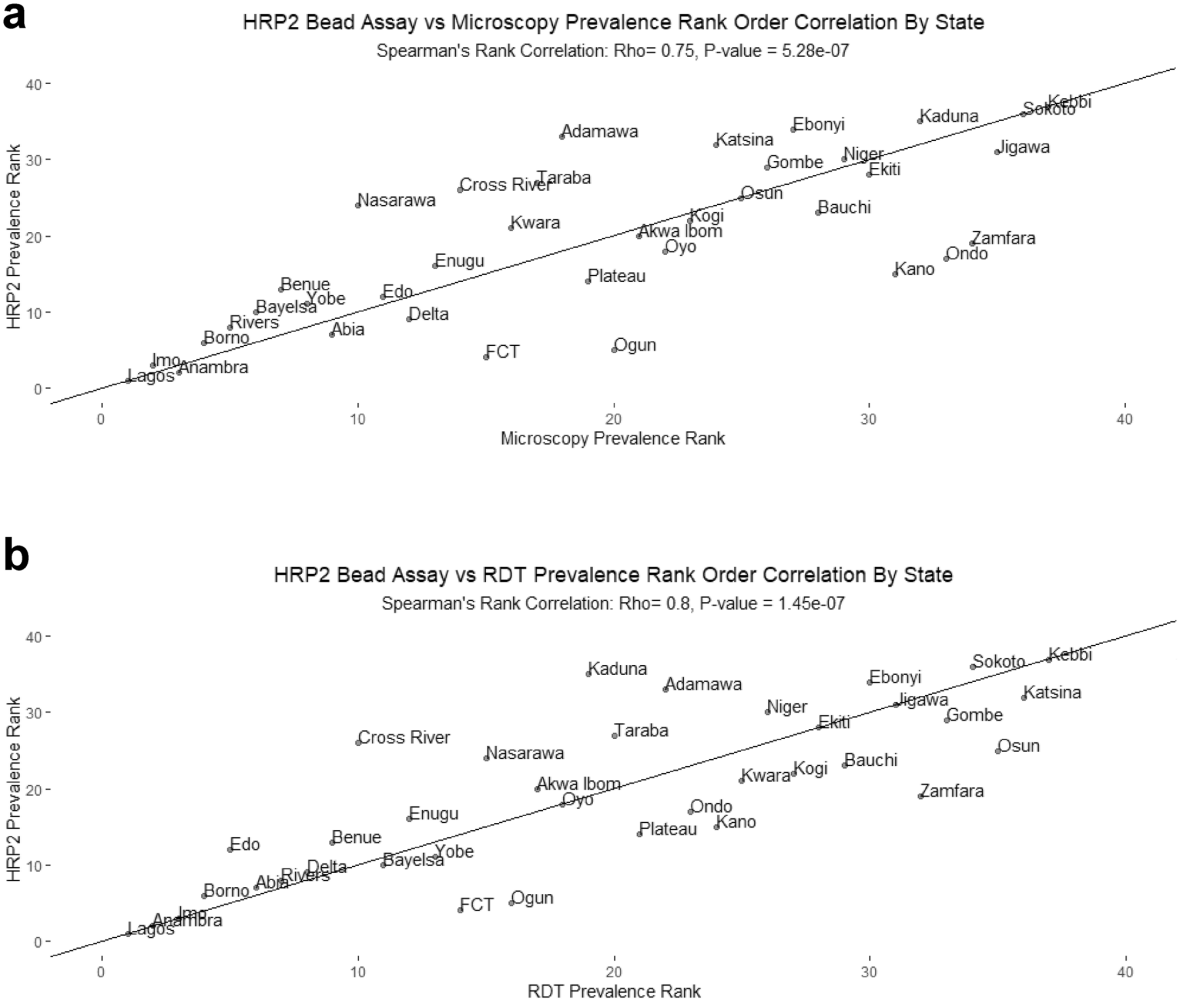
Concordance of prevalence rank correlation by Nigerian state. Panels display scatterplot rank of prevalence by state as well as Spearman’s rank order correlation for (**a**) HRP2 bead assay versus microscopy (Rho = 0.75, p < 0.0001 and (**b**) HRP2 bead assay versus RDT (Rho = 0.80, p < 0.0001). Higher rank denotes higher prevalence. Reference line is x = y.

**Figure 5:**
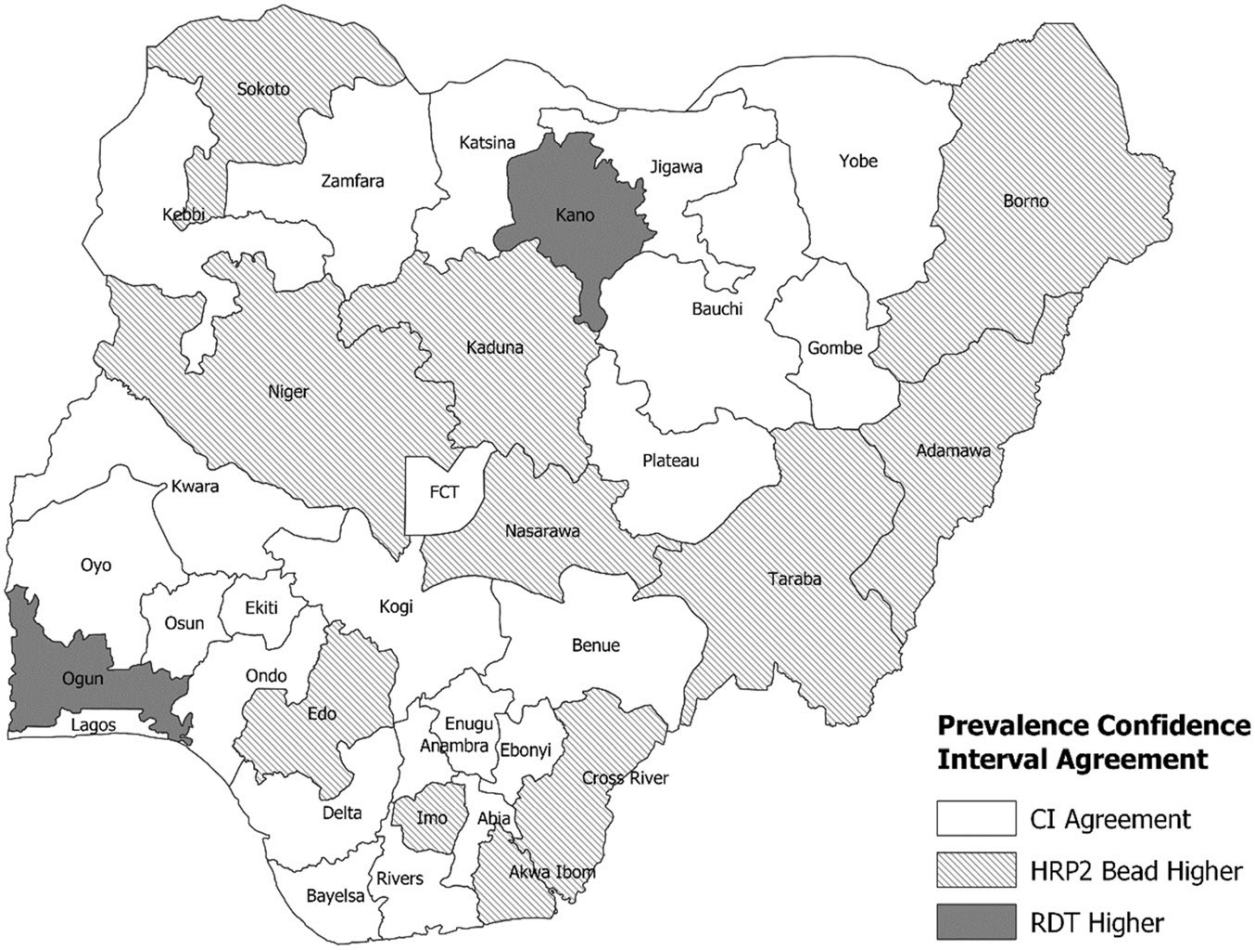
HRP2 bead assay versus RDT prevalence estimate agreement by 95% confidence interval (CI) for each Nigerian state. CI agreement is noted only if HRP2 bead assay prevalence point estimates are within respective RDT prevalence confidence intervals by state and vice versa. Otherwise, the state is classified based on the higher prevalence measure. Map was generated using QGIS version 3.12.2 (www.qgis.org).

Among states, the highest prevalence estimates were seen in Kebbi State: 52.2% (95% CI 43.1–61.3) for microscopy, 76.8% (95% CI 70.1–83.5) for RDT, and 71.8% (95% CI 64.2–79.4) for HRP2 bead assay, while the lowest prevalence estimates were seen in Lagos State: 1.8% (95% CI 0.1–3.4) for microscopy, 3.4% (95% CI 1.3–5.5) for RDT, and 3.5% (95% CI 1.5–5.4) for HRP2 bead assay (Figs. 2, 3). Microscopy prevalence estimates were positively correlated with HRP2 bead assay prevalence estimates by state (Pearson R^2^ = 0.67, p < 0.001) and yielded a statistically significant rank-order correlation (Rho = 0.75, p < 0.001) (Fig. 4a). For the DHS, microscopy and RDT prevalence showed high rank-order correlation as well (Rho = 0.88, p < 0.001) (Supplementary Fig. 1). DHS RDT prevalence estimates were also positively correlated with NMS4 HRP2 bead assay prevalence by state (Pearson R^2^ = 0.71, p < 0.001) and yielded a statistically significant rank-order correlation (Rho = 0.80, p < 0.001) (Fig. 4b). Of 36 Nigerian states plus FCT, between RDT and HRP2 bead assay prevalence estimates, 24 states (64.9%) had overlapping 95% confidence intervals for the two estimates (Fig. 5).

In the DHS, both microscopy and RDT prevalence followed the same temporal trend, with estimates peaking in September with an RDT prevalence of 38.3% (95% CI 34.9–41.6) and declined slightly through December with a prevalence of 35.0% (95% CI 30.6–39.5). Under the NMS4 HRP2 bead assay, prevalence estimates were lowest in July with a prevalence of 29.3% (95% CI 23.3–35.4) and peaked in October with a prevalence of 48.6% (95% CI 45.4–51.8).

## Discussion

Despite having different primary objectives, both the DHS and NAIIS were cross-sectional, two-stage cluster sample, population-based household surveys, yielding national estimates of health and infectious disease indicators in Nigeria during overlapping time periods. While various transmission metrics need to be considered for specific malaria epidemiology contexts, the NAIIS samples tested using the HRP2 bead assay as part of NMS4 yielded overlapping prevalence estimates with the DHS RDT estimates for 65% of all Nigerian states. The high concordance can partially be attributed to the similar design, execution, and timing of the DHS and NAIIS. The similarities between the RDT and HRP2 bead assay estimates ultimately stem from the same underlying parasite biomarker being detected by both tests. During the second half of 2018, there was a true distribution of HRP2 antigen carriage among the Nigerian populace, and both RDTs and the HRP2 bead assay would measure this analyte: the first from capillary blood at the time of participant enrollment (in DHS), and the second in the laboratory from the DBS prepared from blood collected during enrollment (in NAIIS). Therefore, if both sampling designs for DHS and NAIIS were population representative, estimates for the same analyte measured at approximately the same period in a sample from the same population would be expected to be similar. A factor that could result in different prevalence estimates would be if one of the tests was substantially more sensitive than the other. Multiple field and laboratory studies have shown HRP2-based RDTs with good analytical sensitivity, with limits of HRP2 detection typically ranging from 0.40 to 10.0 ng/mL blood^22,23^. The HRP2 bead assay has a limit of quantification of approximately 0.01 ng/mL^24^, but the DBS samples in NMS4 needed to be rehydrated and diluted before performing the assay. For this study, blood samples were diluted 1:40 in the laboratory, meaning that the lowest concentrations of HRP2 from the DBS that would be detected would be approximately 0.40 ng/mL. Assuming good test performance of the SD Bioline Ag P.f. (HRP-II) RDT throughout the DHS, it would then not be surprising that the HRP2 bead assay results from NMS4 gave almost identical prevalence estimates for 65% (24/37) of states, with the HRP2 bead assay providing slightly higher estimates for 30% (11/37) of states and RDT slightly higher estimates for 5.4% (2/37). As expected, DHS microscopy results were slightly lower when compared to RDT as microscopy detects visible blood stage *P. falciparum* parasites versus the HRP2-based RDT detecting an antigen which is abundantly produced and can remain in the blood up to several weeks after an infection is cleared^14,15^. Thus, microscopy will inherently yield a lower but proportional prevalence due to lower diagnostic sensitivity and the more narrow window of detection.

Limitations to this study include discrepancies in the execution of these surveys with differences in response rates and the timing of biomarker collection by state. Overall, the response rate was higher for DHS than NAIIS, including at the household level (99.4% versus 93.9% for DHS and NAIIS, respectively) and at the individual level (96.9% for RDT and 96.3% for microscopy in DHS versus 68.5% among children 0–9 years old in the NAIIS). It is possible that the HIV subject matter as well as the higher volume of blood draw (1 mL capillary blood from children < 2 years and 6 mL venous blood for children 2–14 years versus a few drops from a finger/heel price for DHS participants) resulted in lower response rates for NAIIS compared to DHS. By age group, the lower response rates among NAIIS participants could correlate with the discrepancy in sample size proportions among the lowest age group, where children < 2 years of age represented 34% of all ages in the DHS versus only 16% in the NAIIS. The differential response rates between the two surveys for the youngest age group might have also contributed to the wider difference in prevalence estimates for this group.

Although NAIIS began enrolling participants a month before the start of the DHS (July versus August 2018), the timing of survey administration overlapped between mid-August and through the end of December. Despite this overlap, the surveys differ in the way persons were enrolled by geographic location. Participant enrollment and data collection usually took less than one month per state in the NAIIS instead of the more drawn-out collection in each state in the DHS. Since *P. falciparum* antigen clearance dynamics^14,15^ and the seasonality of malaria transmission led to estimates that are very sensitive to timing, these differences in design—in terms of the start of the surveys, the timing by state, and the duration of data collection by state—could yield discrepancies in estimates between the DHS and NMS4 by state. For example, the combination of an earlier start date and a shorter period of data collection per state resulted in the states of Bauchi, Cross River, Enugu, Kano, Lagos, and Nasarawa States having most of their NAIIS data collection completed before the start of the DHS data collection. Of these, Cross River (21.6 percentage points), Kano (9.1 percentage points), and Nasarawa (14.4 percentage points) states had some of the highest prevalence differences between HRP2 and RDT of all states. National HRP2 bead assay prevalence estimates had larger fluctuations between months compared to RDT estimates and peaked in October (48.6%) compared to September (38.3%) for RDT estimates.

Robust and efficient tools are imperative for accurately estimating malaria prevalence, especially in large-scale heterogeneous settings. Despite the differences in surveys and prevalence measurement tools, we found a high level of agreement between *P. falciparum* antigen prevalence estimates reported from two independent surveys. These estimates also correlated with microscopy results and trends held by various demographic variables. This report provides evidence for utilizing surveys with similar sampling frames for estimation of population-level *P. falciparum* prevalence if the HRP2 antigen is the basis of infection status.

## Methods

### Study area

Both the DHS and NAIIS surveys were administered in the west African country of Nigeria, which is the most populous country in Africa with an estimated population of 196 million in 2018^25^. The country is divided into 36 states and the Federal Capital Territory (FCT) (Fig. 1A), and organized into six geopolitical zones (North East, North West, North Central, South South, South East, and South West). All of Nigeria is endemic for *P. falciparum*, and the 2015 Malaria Indicator Survey estimated prevalence of 45.1% by RDT and 27.4% by microscopy^26^.

### Data sources

#### Demographic and health survey 2018

The DHS provides basic demographic and health indicators in urban and rural areas of each state and the FCT in Nigeria. It was implemented by the National Population Commission of Nigeria in collaboration with the National Malaria Elimination Programme, with funding from various bilateral and multilateral agencies and technical assistance from ICF through the DHS Program.

The sample design was a stratified two-stage cluster sample. Stratification of each state and FCT into rural and urban areas identified 74 sampling strata. Any locality with a population size of 20,000 or more was classified as urban^27^. The 2006 Population and Housing Census of the Federal Republic of Nigeria (NPHC) conducted by the National Population Commission (NPopC) was used as the sampling frame in which wards were subdivided into convenient census enumeration areas (EAs). A combination of cartographic materials demarcating EAs and local government area (LGA) population estimates from the NPHC were used to create a list of EAs and estimate their respective number of households. Sample EAs were selected independently in every stratum. Implicit stratifications were achieved within each strata by sorting the sampling frame before sample selection according to administrative order and using a probability proportional to size selection during the first sampling stage, resulting in 1400 EAs selected.

A household listing exercise was carried out in all selected EAs and served as a sampling frame for selection in the second stage, in which 30 households were selected per EA through equal probability systematic sampling and resulted in a total sample size of approximately 42,000 households without replacement. In one-third (14,000 households) of the sampled 42,000 households, surveyors also collected biomarker information for children aged 6–59 months. Blood samples were taken from finger pricks (or heel prick for children aged 6–11 months) from all children 6–59 months whose parents/guardians consented. Malaria infection was determined using SD Bioline Ag P.f. (HRP-II) RDT (05FK53, Standard Diagnostics, Inc.). Microscopy was performed in 10,500 out of the 14,000 households by thick blood smears. Data were collected between August 14th and December 29th, 2018, with data collection in each state taking place over several weeks or longer.

For inclusion into the study reported here, the household sampled must have been selected for biomarker collection. The household member must have slept in the survey residence the previous night and must have been between the ages of 6–59 months old at the time of the survey.

#### Nigeria HIV/AIDS indicator and impact survey (NAIIS)

NAIIS was a national HIV population-based household survey led by the Government of Nigeria under the Federal Ministry of Health and the National Agency for the Control of AIDS, with technical assistance provided by the U.S. Centers for Disease Control and Prevention (CDC)^21^. The survey was implemented by the NAIIS Consortium, led by the University of Maryland, Baltimore under the supervision of the NAIIS Technical Committee.

The sample design used a stratified two-stage cluster sample. The 37 strata used were the 36 states and FCT. In the first stage, EAs were selected within each state with probabilities proportionate to estimated size according to the projected 2018 number of households based on the 2006 census. State variability of household size was considered when calculating the number of EAs to be selected per state. The resulting sampling frame for NAIIS consisted of 662,855 EAs, of which 4035 were selected. A household listing exercise was carried out in all selected EAs and served as a sampling frame for selection in the second stage. In the second stage, a random sample of 28 households per EA were chosen for inclusion. To generate stable estimates of HIV prevalence at the LGA level in Lagos, the sample size was doubled in Lagos, with eight households sampled per EA. The resulting sampling frame for NAIIS consisted of 28,900,478 households with a sample of 101,580 households selected. Data and samples were collected between July and December 2018, with data collection typically completed in less than one month in each state.

Among sampled households, a subsample consisting of 28,220 households were randomly selected for data and biomarker collection of children aged 0–14. Laboratory staff collected 6 mL venous blood from children aged 2–14 years and 1 mL capillary blood (finger or heel stick) from children aged 6–23 months. Whole blood samples were stored in temperature-controlled cooler boxes. At the end of each day, they were transported to satellite laboratories for processing into plasma and DBS within 24 h of collection. Specimens were stored in − 20 °C freezers and transported to a central laboratory within a week where they were stored in − 80 °C freezers.

Among children < 10 years of age, only those who slept in preselected households the night before (living or visiting) whose parents or guardians were willing and able to provide written consent for participation were included. For children < 10 years of age consent for biomarker testing and consent to future testing was granted by parent or guardian. If the parent or guardian refused receipt of HIV test results, it was considered a refusal and the survey was concluded.

#### Nigeria multi-disease serologic surveillance using stored specimens (NMS4)

The NMS4 project was initiated to conduct serosurveillance using specimens stored with consent and fill knowledge gaps regarding the burden of multiple diseases, including malaria. Initial NMS4 testing focused on testing NAIIS samples from children aged 0 to 14 years and a sample from women of reproductive age.

Whole blood was eluted from a DBS single 6 mm punch (corresponding to 10 µL blood) to provide the sample for the antigen detection assay. The punch was rehydrated in blocking buffer (PBS pH 7.2, 0.5% Polyvinyl alcohol (SigmaAldrich, St. Louis, MO) 0.5% polyvinylpyrrolidine (SigmaAldrich), 0.1% casein (ThermoFisher, Waltham, MA), 0.5% bovine serum albumin (SigmaAldrich), 0.3% Tween-20, 0.05% sodium azide, and 3 µg/mL *E. coli* extract to prevent non-specific binding) to a final dilution of 1:40. Eluted blood samples were stored at 4 °C until assayed.

HRP2 antigen detection was performed by a bead-based multiplex assay as described previously^24^. Assay plates were read on MAGPIX™ machines (Luminex Corp, Austin, TX), and at a target of 50 beads/region, the median fluorescence intensity (MFI) was generated for the HRP2 analyte. To determine the assay signal threshold for antigen positivity, a finite mixture model approach was employed to estimate the ‘antigen negative’ population within these data (using data from specimens from all children < 15 years of age from 36 states plus FCT plus the sample of 9919 women of reproductive age with completed antigen testing) and then establishing a cutoff using the mean + 2 standard deviations from this distribution^28,29^.

### Statistical information

Data cleaning and analysis were performed using R (Version 4.0.2). Prevalence estimates and confidence intervals accounting for the complex survey designs were calculated using the R “survey” package (Version 4.0). All prevalence estimates in this report are weighted for probability of selection and non-response, while all counts are unweighted unless otherwise specified. For this analysis, only children aged 6–59 months who had slept in the residence the previous night were included from NAIIS, to mirror the population included in DHS. Spearman’s rank-order correlation test was used to estimate concordance between the rank-ordered prevalence by state from the two surveys and was calculated using the package “stats” as part of the base R platform. Maps were generated using QGIS (Version 3.12.2-Bucureşti).

### Ethical approval

For the DHS, blood samples were taken from finger pricks (or heel prick for children aged 6–11 months) from all children 6–59 months whose parents/guardians consented. For NAIIS, for children < 10 years of age consent for biomarker testing and consent to future testing was granted by parent or guardian. All methods were carried out in accordance with relevant guidelines and regulations. For enrollment into either survey, informed consent was obtained from each child’s legal guardian. For the NAIIS, human subjects review was conducted by the IRBs of the U.S. Centers for Disease Control and Prevention (CDC), University of Maryland, Baltimore, and the Nigerian Health Research Ethics Committee (NHREC). All experimental protocols for NMS4 were approved by the Government of Nigeria under the Federal Ministry of Health and the CDC. The survey protocol for DHS was reviewed and approved by NHREC.

## Supplementary Information

Supplementary Information.

## Data Availability

DHS data are publicly available for download. NMS4 data are owned by the Government of Nigeria (GoN); requests for NMS4 data must be approved by GoN and all other NMS4 principal investigators. Inquiries should be directed to the corresponding author.
