# Supplementary material for: Plasmodium falciparum infection prevalence among children aged 6–59 months from independent DHS and HIV surveys: Nigeria, 2018
Source: Sci Rep. 2023 Feb 3;13:1998. doi: 10.1038/s41598-023-28257-0 (PMC9898257; doi:10.1038/s41598-023-28257-0)

**Supplementary Information**

**Supplementary Table 1. Unweighted Participant Count of 6-59-Month-Old Children in Nigeria Between DHS and NMS4 Among Demographic and Geographic Variables.**

|  |  | **DHS** | |  | **NMS4** |
| --- | --- | --- | --- | --- | --- |
|  |  | **Microscopy** | **RDT** |  | **HRP2 Bead Assay** |
|  |  | **N (%)** | **N (%)** |  | **N (%)** |
| Total |  | 8,127 (100%) | 11,173 (100%) |  | 8,029 (100%) |
| Sex |  |  |  |  |  |
| Female |  | 3,999 (49.2%) | 5,523 (49.4%) |  | 3,912 (48.7%) |
| Male |  | 4,128 (50.8%) | 5,650 (50.6%) |  | 4,117 (51.3%) |
| Age in Months |  |  |  |  |  |
| 6-23 |  | 2,720 (33.5%) | 3,748 (33.5%) |  | 1,249 (15.6%) |
| 24-35 |  | 1,757 (21.6%) | 2,401 (21.5%) |  | 1,913 (23.8%) |
| 36-47 |  | 1,823 (22.4%) | 2,485 (22.2%) |  | 2,277 (28.4%) |
| 48-59 |  | 1,827 (22.5%) | 2,539 (22.7%) |  | 2,590 (32.3%) |
| Month of Collection |  |  |  |  |  |
| July |  | 0 (0.0%) | 0 (0.0%) |  | 611 (7.6%) |
| August |  | 558 (6.9%) | 810 (7.2%) |  | 1,312 (16.3%) |
| September |  | 2,325 (28.6%) | 3,174 (28.4%) |  | 1,588 (19.8%) |
| October |  | 2,058 (25.3%) | 2,810 (25.1%) |  | 1,698 (21.1%) |
| November |  | 1,787 (22.0%) | 2,470 (22.1%) |  | 1,734 (21.6%) |
| December |  | 1,399 (17.2%) | 1,909 (17.1%) |  | 1,086 (13.5%) |
| State |  |  |  |  |  |
| North Central |  | 1,417 (17.4%) | 1,973 (17.7%) |  | 1,181 (14.7%) |
| Benue |  | 236 (2.9%) | 339 (3.0%) |  | 264 (3.3%) |
| FCT Abuja |  | 199 (2.4%) | 269 (2.4%) |  | 55 (0.7%) |
| Kogi |  | 140 (1.7%) | 194 (1.7%) |  | 112 (1.4%) |
| Kwara |  | 181 (2.2%) | 244 (2.2%) |  | 116 (1.4%) |
| Nasarawa |  | 199 (2.4%) | 265 (2.4%) |  | 122 (1.5%) |
| Niger |  | 256 (3.1%) | 371 (3.3%) |  | 323 (4.0%) |
| Plateau |  | 206 (2.5%) | 291 (2.6%) |  | 189 (2.4%) |
| North East |  | 1,436 (17.7%) | 2,023 (18.1%) |  | 1,267 (15.8%) |
| Adamawa |  | 215 (2.6%) | 281 (2.5%) |  | 180 (2.2%) |
| Bauchi |  | 244 (3.0%) | 392 (3.5%) |  | 352 (4.4%) |
| Borno |  | 238 (2.9%) | 330 (3.0%) |  | 140 (1.7%) |
| Gombe |  | 261 (3.2%) | 372 (3.3%) |  | 230 (2.9%) |
| Taraba |  | 230 (2.8%) | 311 (2.8%) |  | 202 (2.5%) |
| Yobe |  | 248 (3.1%) | 337 (3.0%) |  | 163 (2.0%) |
| North West |  | 1,876 (23.1%) | 2,605 (23.3%) |  | 2,230 (27.8%) |
| Jigawa |  | 287 (3.5%) | 380 (3.4%) |  | 353 (4.4%) |
| Kaduna |  | 280 (3.4%) | 376 (3.4%) |  | 481 (6.0%) |
| Kano |  | 359 (4.4%) | 510 (4.6%) |  | 395 (4.9%) |
| Katsina |  | 305 (3.8%) | 439 (3.9%) |  | 406 (5.1%) |
| Kebbi |  | 218 (2.7%) | 318 (2.8%) |  | 250 (3.1%) |
| Sokoto |  | 186 (2.3%) | 259 (2.3%) |  | 234 (2.9%) |
| Zamfara |  | 241 (3.0%) | 323 (2.9%) |  | 111 (1.4%) |
| South East |  | 898 (11.0%) | 1,246 (11.2%) |  | 1,075 (13.4%) |
| Abia |  | 182 (2.2%) | 263 (2.4%) |  | 170 (2.1%) |
| Anambra |  | 308 (3.8%) | 395 (3.5%) |  | 242 (3.0%) |
| Ebonyi |  | 350 (4.3%) | 444 (4.0%) |  | 192 (2.4%) |
| Enugu |  | 187 (2.3%) | 243 (2.2%) |  | 178 (2.2%) |
| Imo |  | 235 (2.9%) | 319 (2.9%) |  | 293 (3.6%) |
| South South |  | 1,262 (15.5%) | 1,664 (14.9%) |  | 1,148 (14.3%) |
| Akwa Ibom |  | 161 (2.0%) | 233 (2.1%) |  | 225 (2.8%) |
| Bayelsa |  | 195 (2.4%) | 259 (2.3%) |  | 115 (1.4%) |
| Cross River |  | 97 (1.2%) | 140 (1.3%) |  | 160 (2.0%) |
| Delta |  | 133 (1.6%) | 181 (1.6%) |  | 226 (2.8%) |
| Edo |  | 104 (1.3%) | 146 (1.3%) |  | 182 (2.3%) |
| Rivers |  | 208 (2.6%) | 287 (2.6%) |  | 240 (3.0%) |
| South West |  | 1,238 (15.2%) | 1,662 (14.9%) |  | 1,128 (14.0%) |
| Ekiti |  | 186 (2.3%) | 254 (2.3%) |  | 97 (1.2%) |
| Lagos |  | 252 (3.1%) | 321 (2.9%) |  | 402 (5.0%) |
| Ogun |  | 202 (2.5%) | 278 (2.5%) |  | 153 (1.9%) |
| Ondo |  | 156 (1.9%) | 209 (1.9%) |  | 116 (1.4%) |
| Osun |  | 178 (2.2%) | 251 (2.2%) |  | 95 (1.2%) |
| Oyo |  | 264 (3.2%) | 349 (3.1%) |  | 265 (3.3%) |
|  |  |  |  |  |  |

**Supplementary Figure 1. DHS RDT versus Microscopy.** Prevalence estimates by state are sorted in descending order of RDT positivity in comparison with microscopy prevalence (A), and the correlation between the two tests (B).


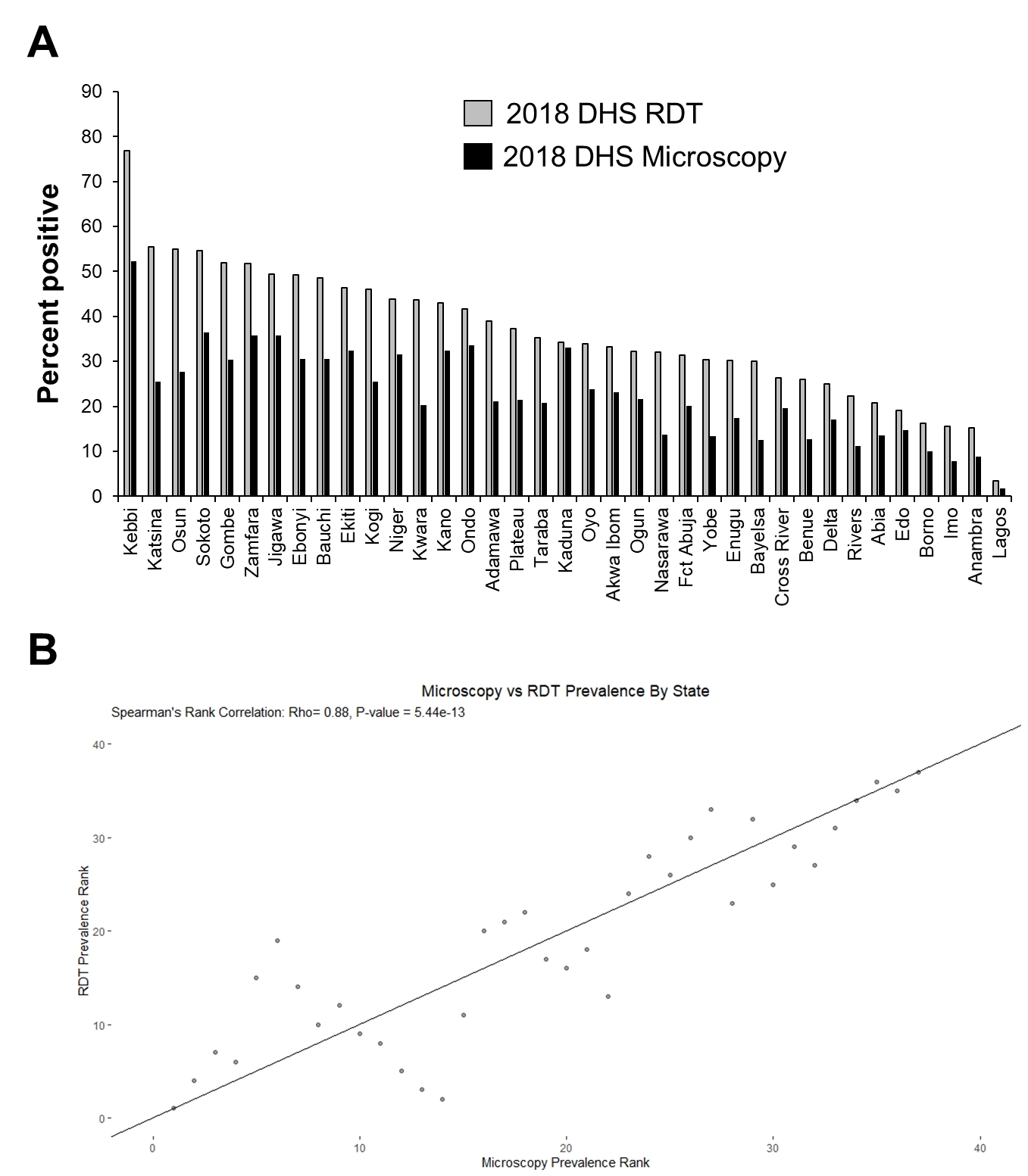

Supplement: Supplementary file 1 — Supplementary Information. [file 41598_2023_28257_MOESM1_ESM.docx]
